# Characterization of juvenile play in rats: importance of sex of self and sex of partner

**DOI:** 10.1186/s13293-015-0034-x

**Published:** 2015-09-10

**Authors:** Kathryn J. Argue, Margaret M. McCarthy

**Affiliations:** Department of Pharmacology and Program in Neuroscience, University of Maryland School of Medicine, 655 W Baltimore St, Baltimore, MD 21201 USA

**Keywords:** Social play behavior, Juvenile, Sex differences, Play partner

## Abstract

**Background:**

Juvenile social play is observed in many mammalian species, and its disruption in several neuropsychiatric disorders has greatly increased interest in understanding the origins and sources of variability in this behavior.

**Methods:**

We quantified social play behavior in juvenile rats and investigated the impact of sex and familiarity of the play partner. Sex differences in play behavior were investigated by comparing males and females from either same- or mixed-sex pairs with data pooled over 12 days of analysis. Whether play was altered based on the sex of the play partner was assessed using a paired analysis to compare play with a same- or opposite-sex play partner for both males and females. Additionally, a repeated measures design was utilized to determine whether play changed with increasing age. On postnatal day 33, a novel play partner was introduced. We used a repeated measures analysis to compare postnatal day 33 with the previous day. These approaches were used to assess the effects of age, sex, sex of partner, and familiarity of partner on total social play behavior as well as how play was broken down into components, such as pouncing, pinning, chasing, and boxing.

**Results:**

There were sex differences in total frequency of play, and specific parameters of play behavior, such as chasing, pouncing, pinning, and boxing. Additionally, males significantly altered their play behavior in response to the sex of their play partner, whereas females were more sensitive to the familiarity of the play partner.

**Conclusions:**

This study provides critical groundwork for uncovering factors that regulate social play behavior and can be used to guide future mechanistic based work.

## Background

Juvenile social play behavior is ubiquitous across a variety of mammalian species (for example [1, 2]). Rough-and-tumble play, or play fighting, is the most common form of play in many species, including rats [3–6]. Play is a complex behavior, requiring both the ability to initiate social interactions and to respond appropriately to social cues from others. Recently, there has been a resurgence in the investigation of social behaviors due to their disruption in several neuropsychiatric disorders, such as schizophrenia, autism, and attention-deficit hyperactivity disorder [7–10]. Despite its frequent occurrence and perturbation in illness, there is continued debate regarding the purpose of social play. Studies in children with disabilities and in animals that have been deprived of play during the juvenile period indicate that social play is critical for normal social, motor, cognitive, and emotional development [1, 2, 11–15].

In many species, there is a higher frequency of rough-and-tumble play events by males, relative to females (i.e., [16–25]). However, there are several reports that the sex difference in the social play of rats is an artifact of methodology due to dependency on strain, isolation protocol, home cage versus neutral cage, and the number and sex of play partners [16, 21]. For those studies that do report a sex difference, the incidence is often limited to the initiation of play, although more detailed studies demonstrate that there are quantitative as well as qualitative sex differences in play behavior [26]. Several of the brain areas critical for the expression of social play behavior, which include cortical, limbic, hypothalamic, thalamic, and sensory areas [27, 28], are responsive to sex steroid hormones [29, 30]. The medial amygdala in particular is considered critical for sexual differentiation of play behavior because implantation of testosterone capsules into this region is sufficient to masculinize the frequency of female social play behavior [31]. In further support of amygdalar mediation of sex-specific play behavior, Olesen et al. [32] demonstrated that ligand-independent activation of estrogen receptors by dopamine specifically within the central amygdala and bed nucleus of the stria terminalis can masculinize female social play behavior.

We performed a detailed analysis of social play behavior in Sprague-Dawley rats, which are known to exhibit sex differences in this behavior, with some reporting higher rates of play in males [18], while others report higher rates in females [26] or no sex difference in initiation of play [33]. Several studies have provided evidence for the contagiousness of play [26, 34–38], making it difficult to determine whether differences in play (or the lack therefore) in studies performed with mixed sex or treatment groups are genuine or an artifact of the group dynamic. To shed light on the impact of methodological differences on the reporting of sex differences in rough-and-tumble play behavior, we assessed how the sex and familiarity of the play partner can influence specific aspects of play behavior. For these experiments, all animals were assessed playing with both a same- and an opposite-sex partner so that comparisons on how the sex of the play partner altered both the time spent playing and the frequency of play behaviors could be assessed with a repeated measures design. All experiments were carried out without prior social isolation, a method commonly used to increase play behavior, to emphasize that sex differences in play behavior are observed under basal conditions. Testing was conducted on postnatal day (PN) 27–38, an age when juveniles demonstrate the full repertoire of play behaviors [5]. We assessed the frequency of play events and time spent engaged in play, as well as how play was broken into its constitutive components of chasing, pouncing, pinning, and boxing. Various aspects of play behavior, such as offensive versus defensive behaviors and discrimination of play partner, are mediated by different regions of the brain, making it is feasible to alter specific parameters while leaving others unaffected (for example, see [39]). It was of particular interest in this study to examine individual aspects of play behavior because a recent study comparing sex and strain differences in rough-and-tumble play behavior following social isolation reported sex differences in defensive strategies in play behavior with no differences in total frequency of play or play initiation in the Sprague-Dawley strain [33]. Understanding sex differences in social play behavior provides an important step towards revealing factors that mediate social play and how it may be disrupted in neuropsychiatric disorders.

## Methods

### Animals

Sprague-Dawley rats (Harlan) mated in our facility were allowed to deliver normally under standard laboratory conditions. On the day of birth (PN0), three litters were culled to 12 pups consisting of equal numbers of males and females. Pups were weaned on PN22 and housed in groups consisting of two to three individuals of the same sex, with each cagemate from a different litter. The animals were housed in polycarbonate cages (20 × 40 × 20 cm) with corncob bedding under a reverse 12:12 h light/dark cycle. Food and water were available ad libitum. All breeding and experimental procedures were approved by the Institutional Care and Use Committee at the University of Maryland, Baltimore, and performed in accordance with national animal care and use guidelines.

### Play behavior

Play behavior was assessed under red-light illumination during the dark phase of the cycle in a neutral arena (49 × 37 × 24 cm) with TEK-Fresh cellulose bedding (Harlan Laboratories) to which the pups were habituated by being allowed to explore for 10 min with their cagemates on PN26. Experiments began at a minimum of 2 h after lights off. A neutral arena was chosen to facilitate assessment of different play partners, as opposed to cagemates. Play behavior was assessed everyday from PN27–PN38. Prior to behavioral assessment, pups were given head and tail markings to distinguish individuals. On each day, individuals were studied during a play session with a same-sex partner and another session with an opposite-sex partner, with 2 h between play sessions. The order of the pairs was alternated to eliminate any effect of which play partner was encountered first. Pups were paired with the same two play partners every day except for PN33, on which both the same- and opposite-sex partners were switched for two novel play partners to test the effects of a novel versus a familiar play partner. All play partners were age-matched and were not litter- or cagemates. Each play session consisted of a 2-min acclimation period, followed by a 10-min video recorded session that was played back for analysis. Following play, pups were returned to their home cage. During analysis, the frequency of pouncing, pinning, chasing, and boxing behaviors were scored for each individual and the total time engaged in play was scored for each pair (*n* = 6 males and 6 females from three different litters).

### Weight

Weight was recorded every other day from PN28–PN38 (*n* = 4 males and 5 females from three different litters).

### Statistics

Total frequency of play and frequency of pouncing, pinning, chasing, and boxing were analyzed by combining results from all days of analysis. We first tested for sex differences in same- or mixed-sex pairs using Student’s *t* test with Welch’s correction. We then tested for an effect of the sex of the play partner using a paired *t* test to analyze the behavior of males and females in same-sex pairs relative to their behavior in mixed-sex pairs. For each parameter of play behavior, data was separated by age and analyzed using a two-way ANOVA with Tukey post hoc analysis with age as a repeated measure and pair type as the other factor to determine whether play differed with increasing age in a pair-dependent manner. This analysis also serves to identify potential sources of variability in our initial analysis where the data was pooled across all ages. To determine how the switch from a familiar to a novel play partner affected play, we used a two-way ANOVA with Bonferroni post hoc analysis with partner familiarity as a repeated measure and pair type as the other factor. Sex differences in weight were assessed using repeated measures ANOVA with Bonferroni post hoc analysis. Percent change in weight between the sexes was also calculated. For all analyses, differences were considered significant when *p* < 0.05. All data are expressed as mean or mean with standard error of the mean.

## Results

### Sex differences in the total frequency of rough-and-tumble play behavior

**Fig. 1:**
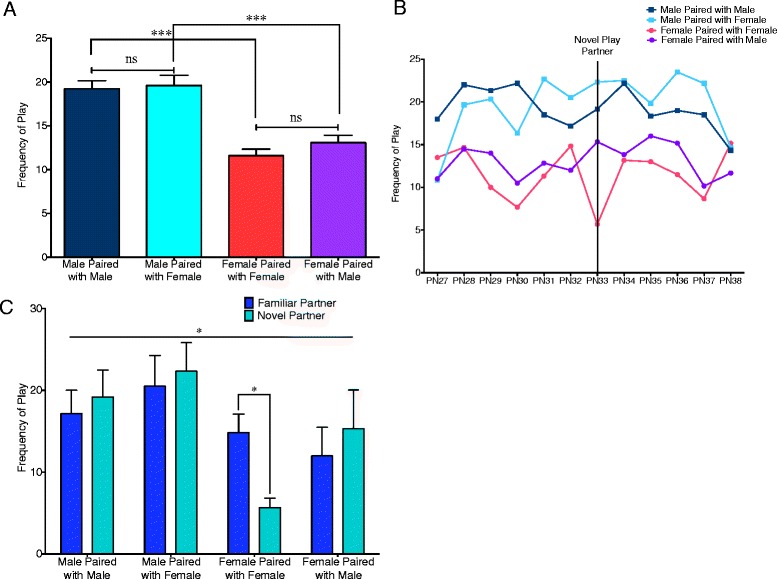
Males play more than females. Male and female pups were assessed for play behavior on PN27–PN38 with a same- and an opposite-sex partner. Play partners were the same every day, except for PN33, on which novel play partners were introduced. **a** Combining frequency of play from PN27–PN38 showed sex differences in both same- and mixed-sex pairs (Student’s *t* tests with Welch’s correction: ***p < 0.001). However, sex of the play partner did not change total frequency of play (paired *t* tests: ns = not significant). **b** Separation of play behavior into individual days did not indicate an interaction between the pair type and age (two-way repeated measures ANOVA). **c** Comparison of play on PN32 (familiar partner) and PN33 (novel partner) revealed a reduction in play for females paired with novel female partners (two-way repeated measures ANOVA with Bonferroni post hoc: *p < 0.05)

Total frequency of all rough-and-tumble play behaviors between males and females paired with either same- or opposite-sex play partners from PN27–PN38 was analyzed using *t* tests to test for differences between males and females and using paired *t* tests to test for effects of the sex of the play partner (Fig. 1a). Both same- and mixed-sex pairs showed significant sex differences in which males displayed higher frequencies of play behavior related to females (*t*(135.5) = 6.430, *p* < 0.0001 and *t*(129.1) = 4.574, *p* < 0.001, respectively). Neither males nor females demonstrated a significant change in the total frequency of play behaviors when paired with a same- versus opposite-sex play partner (*t*(71) = 0.3874, *p* = 0.6998 for males; *t*(71) = 1.441, *p* = 0.1541 for females). A two-way ANOVA comparing each of the pair types with age as a repeating factor did not show a significant effect of age (*F*(11,220) = 1.370, *p* = 0.1885) (Fig. 1b). To assess the impact of familiarity of the partner, play on PN32 (familiar partner) was compared to play on PN33 (novel partner). There was a significant interaction between the pair type and familiarity of the play partner (*F*(3,20) = 3.961, *p* = 0.0228) with the Bonferroni post-test indicating a significant decrease in females paired with other females with the introduction of a novel partner (*t*(20) = 3.136, *p* = 0.028) (Fig. 1c). Because there was some variability in the frequency of play in the female-female dyads across the days, the frequency of play on the days before and after the introduction of the novel partner (PN27–PN32, PN34–PN38) was averaged and compared to PN33 using a *t* test with Welch’s correction (*t*(10.07) = 4.610, *p* = 0.0009). This ensures that the observed decrease in female-female dyads with the introduction of a novel partner was not an artifact of elevated play levels on PN32 relative to other days.

### Sex, play partner, and age altered the frequency of pouncing behavior

**Fig. 2:**
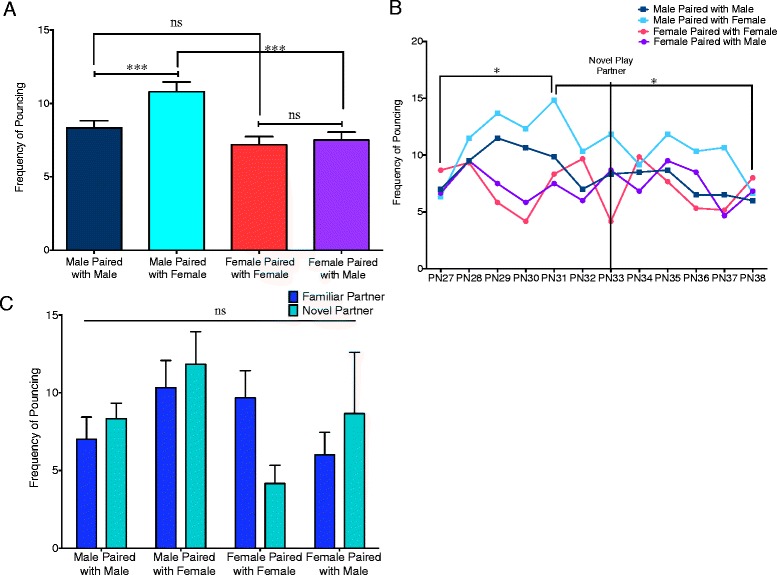
Pouncing frequency is affected by sex, play partner, and age*.* **a** Males pounced more than females when in mixed-sex pairs, but not in same-sex pairs (Student’s *t* tests with Welch’s correction: ***p < 0.001, ns = not significant). Males increased their pouncing frequency when paired with a female relative to when paired with another male (paired *t* tests: ***p < 0.001). There was no change in female pouncing behavior dependent on the sex of her play partner (paired *t* test: ns = not significant). **b** Separation of pouncing behavior into individual days revealed a significant main effect of age (two-way repeated measures ANOVA) with Tukey post hoc analysis revealing increased pouncing frequency in males paired with females on PN31 relative to both PN27 and PN38 (*p < 0.05). **c** Comparison of play on PN32 (familiar partner) and PN33 (novel partner) did not reveal an interaction between pair type and partner familiarity (two-way repeated measures ANOVA: ns = not significant)

Pouncing is considered one of the most characteristic parameters of play behavior in the rat. It occurs when one rat jumps on the other in an attempt to gain access to the nape of the neck. The frequency of pouncing behavior was analyzed for males and females with same- and opposite-sex partners with the data from all ages pooled together (Fig. 2a). Males and females paired with same-sex partners did not display significant sex differences (*t*(139.4) = 1.563, *p* = 0.1203); however, males and females paired with opposite-sex partners did display a sex difference with males pouncing more than females (*t*(136.9) = 3.772, *p* = 0.0002). This discrepancy in the sex differences observed with same- versus opposite-sex pairs was due to an increase in pouncing when males were paired with females relative to males paired with other males (*t*(71) = 3.591, *p* = 0.0006). Conversely, females did not alter their frequency of pouncing depending on the sex of their play partner (*t*(71) = 0.4841, *p* = 0.6298). A two-way ANOVA comparing each of the pair types with age as a repeating factor showed a main effect of age (*F*(11,220) = 1.879, *p* = 0.0433) (Fig. 2b). Tukey post-test analysis showed significant differences only in the males paired with females group in which pouncing was increased on PN31 relative to PN27 and PN38 (*p* < 0.05 for both). Comparison of pouncing on PN32 (familiar partner) and PN33 (novel partner) to test for the effect of partner familiarity did not reveal a significant interaction between pair type and partner familiarity (*F*(3,20) = 2.157, *p* = 0.1250) (Fig. 2c).

### Sex of self and sex of play partner affected frequency of pinning

**Fig. 3:**
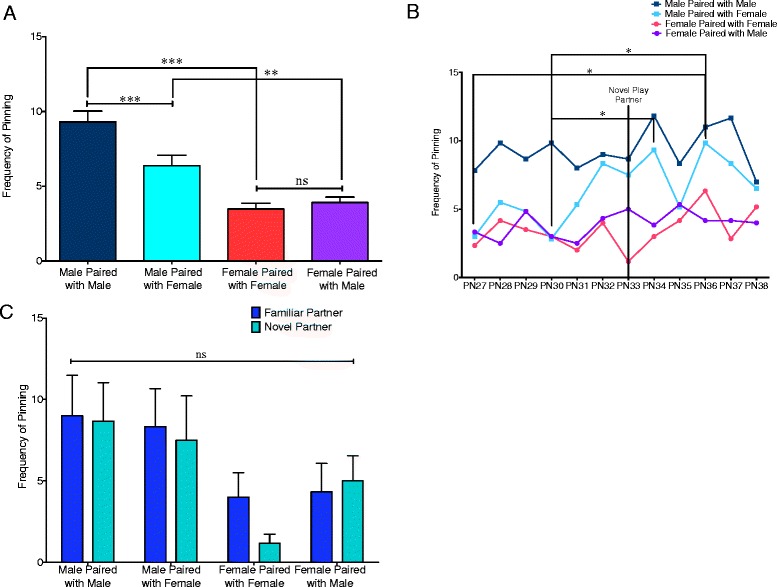
Pinning frequency is modulated by play partner, sex, and age. **a** Males exhibited a higher pinning frequency relative to females in both same- and mixed-sex pairs (Student’s *t* tests with Welch’s correction: **p < 0.01, ***p < 0.001). Males decreased their pinning frequency when paired with a female relative to when paired with another male, while frequency of female pinning was not dependent on the sex of her play partner (paired *t* tests: ***p < 0.001, ns = not significant). **b** Separation of pinning behavior into individual days revealed a significant main effect of age (two-way repeated measures ANOVA) with Tukey post hoc analysis revealing differences in males paired with females on PN27 and PN30 relative to PN36 and on PN30 relative to PN34 (*p < 0.05). **c** Comparison of play on PN32 (familiar partner) and PN33 (novel partner) did not reveal an interaction between pair type and partner familiarity (two-way repeated measures ANOVA: ns = not significant)

Rough-and-tumble play is comprised of a combination of attack and defense maneuvers. During the juvenile period, rotating to supine is the most common defense strategy [6]. This maneuver places one animal lying on its back with the other standing on top, in a pinning position. From the supine position, the “pinned” animal can block access to the nape of the neck, the major target of attack in play fighting. A decrease in pinning behavior may be due to a decrease in the number of attacks launched, a decreased response to play solicitation, or a combination of the two. Analyses of pinning frequency for males and females with same- and opposite-sex partners from PN27–PN38 identified sex differences between both same- and mixed-sex pairs (*t*(107.8) = 7.073, *p* = 0.0001 and *t*(106.3) = 3.117, *p* = 0.0024, respectively) (Fig. 3a). A paired *t*-test showed that males decrease their pinning frequency when paired with females compared to when paired with other males (*t*(71) = 4.062, *p* = 0.001), while female pinning frequency was comparable with same- and opposite-sex play partners (*t*(71) = 0.9228, *p* = 0.3592) (Fig. 3a). A two-way ANOVA using age as a repeated measure and pair type revealed a significant main effect of age (*F*(11,220) = 2.525, *p* = 0.0051) (Fig. 3b). Tukey post-test analysis demonstrated a significant increase in pinning on PN36 relative to both PN27 and PN30 as well as a significant increase on PN34 relative to PN30 in the male paired with female group (*p* < 0.05 for all). Analysis of pinning behavior on PN32 (familiar partner) relative to PN33 (novel partner) did not reveal a significant interaction between pair type and partner familiarity (*F*(3,20) = 0.7685, *p* = 0.5251) (Fig. 3c).

### Sex of play partner affected chasing frequency

**Fig. 4:**
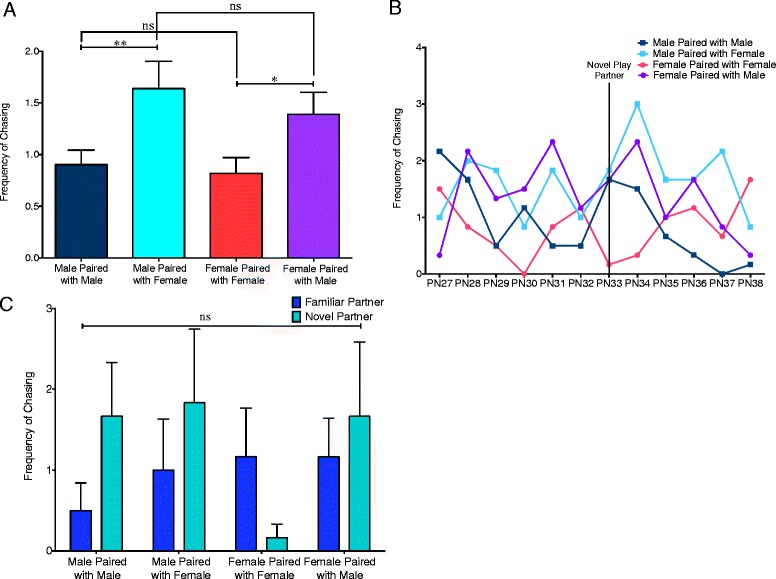
Chasing is affected by the sex of the play partner. **a** There were no sex differences in either same- or mixed-sex pairs in frequency of chasing behavior (Student’s *t* tests with Welch’s correction: ns = not significant). However, both males and females increased their chasing when in mixed- relative to same-sex pairs (paired *t* tests: *p < 0.05, **p < 0.01). **b** Separation of chasing behavior into individual days did not reveal a significant interaction between age and pair type (two-way repeated measures ANOVA). **c** Comparison of play on PN32 (familiar partner) and PN33 (novel partner) did not reveal an interaction between pair type and partner familiarity (two-way repeated measures ANOVA: ns = not significant)

Chasing occurs when one pup (the attacker) runs after another (the evader), which will either result in successful escape by the evader, the attacker catching the opponent and performing either a pounce or pin, or for the chase to become circular in which both pups chase one another. In some studies, chasing is considered a type of locomotor play or social play that is separate from rough-and-tumble play because it does not involve direct contact between the two animals. We chose to include it in our analysis because it can be indicative of defensive tactics, such as evasion, used during rough-and-tumble play. PN27–PN38 males and females with either same- or opposite-sex partners did not display sex differences in frequency of chasing behavior (*t*(141) = 0.4026, *p* = 0.6878 and *t*(136.1) = 0.7336, *p* = 0.4645, respectively) (Fig. 4a). However, there was a significant effect with the sex of the play partner in which both males and females increased their chasing behavior when paired with an opposite-sex partner relative to when paired with a same-sex partner (*t*(71) = 2.700, *p* = 0.0087 for males and *t*(71) = 2.249, *p* = 0.0433 for females). For frequency of chasing, there was no interaction between age and pair type (*F*(33,220) = 1.201, *p* = 0.2194) (Fig. 4b) and no interaction between pair type and partner familiarity (*F*(3, 220) = 0.8365, *p* = 0.4897 (Fig. 4c).

### Sex affected frequency of boxing

**Fig. 5:**
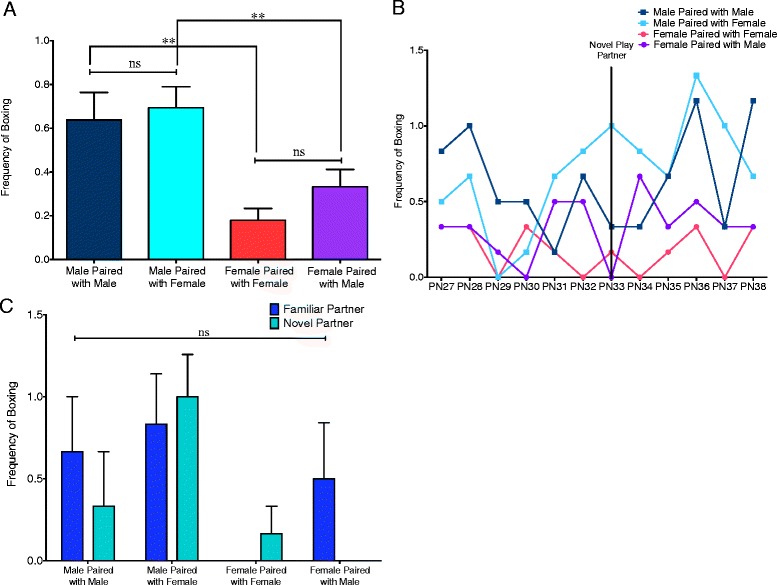
Males show higher boxing frequency. **a** Males displayed a higher frequency of boxing behavior in both same- and mixed-sex pairs (Student’s *t* tests with Welch’s correction: **p < 0.01). There were no significant changes in frequency of boxing behavior dependent on the sex of the play partner (paired *t* tests: ns = not significant). **b** Separation of boxing behavior into individual days did not reveal a significant interaction between age and pair type (two-way repeated measures ANOVA). **c** Comparison of play on PN32 (familiar partner) and PN33 (novel partner) did not reveal an interaction between pair type and partner familiarity (two-way repeated measures ANOVA: ns = not significant)

Overall boxing was the most infrequent behavior, but sex differences were observed in both same- and mixed-sex pairs (*t*(95.99) = 3.357, *p* = 0.0011 and *t*(136.9) = 2.900, *p* = 0.0044, respectively) (Fig. 5a). Additionally, neither males nor females altered their boxing behavior when paired with a same- relative to opposite-sex partners (*t*(71) = 34.60, *p* = 0.7304 for males and *t*(71) = 1.555, *p* = 0.1243 for females) (Fig. 5a). There was no interaction between age and pair type (*F*(33,220) = 0.6879, *p* = 0.9005 (Fig. 5b) or pair type and partner familiarity (*F*(3,22) = 0.8585, *p* = 0.4786) (Fig. 5c) for this parameter.

### Play partner affected the time spent engaged in play

**Fig. 6:**
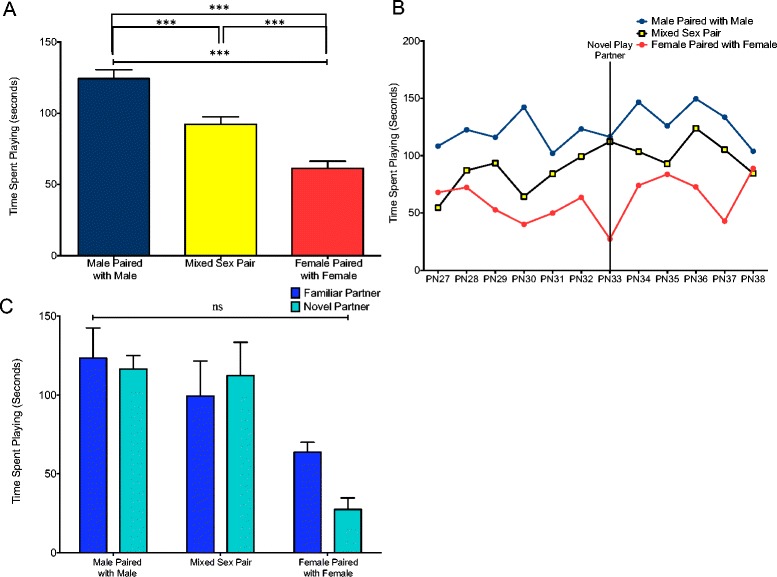
Males play longer than females. **a** The time spent engaged in play was greatest for males paired with other males and least for females paired with other females, with mixed-sex pairs demonstrating intermediate levels (ANOVA: ***p < 0.001). **b** Separation of the time spent engaged in play into individual days did not reveal a significant interaction between age and pair type (two-way repeated measures ANOVA). **c** Comparison of play on PN32 (familiar partner) and PN33 (novel partner) did not reveal an interaction between pair type and partner familiarity (two-way repeated measures ANOVA: ns = not significant)

An ANOVA of the time each pair spent engaged in play behavior from PN27–PN38, showed a significant difference amongst the three pairings (*F*(2,141) = 22.23, *p* = 0.0001) (Fig. 6a). Tukey post-test analysis demonstrated that male same-sex pairs spent more time engaged in play than either mixed-sex or female same-sex pairs and that female same-sex pairs spent the least amount of time playing; differences between all three groups were significant at the *p* < 0.001 level. There was no interaction between pair type and age (*F*(22,99) = 0.9820, *p* = 0.4932) (Fig. 6b) and no interaction between familiarity of partner and pair type (*F*(2,9) = 0.8604, *p* = 0.4551) (Fig. 6c) for this parameter.

### Relationship dynamics within the pairs were not always stable

**Fig. 7:**
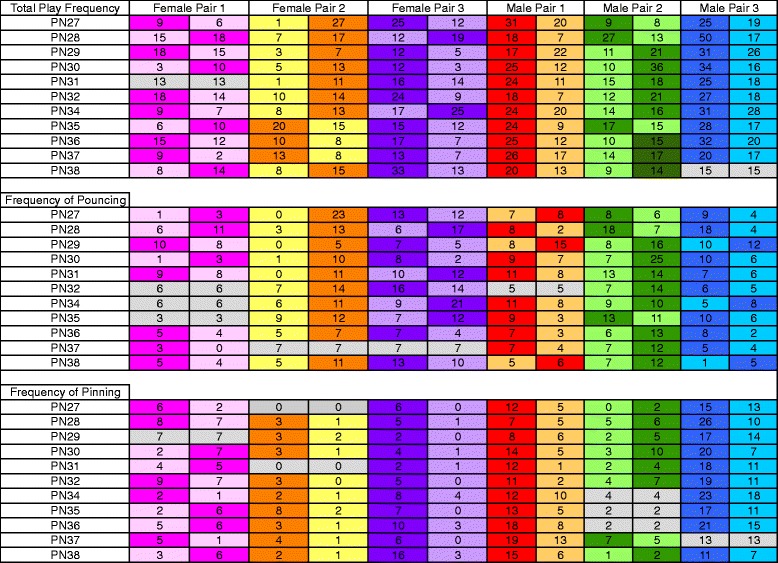
Relative playfulness is dynamic within pairs. Each column indicates a single individual from a same-sex pair set. Age is indicated in the far *left column*. PN33, on which a novel play partner was introduced, was excluded. For each pair, the individual displaying the higher frequency is indicated in a darker shade for either total play (cumulative pouncing, pinning, boxing and chasing), and two individual play components, pouncing and pinning. The individual displaying the lower frequency of each component is indicated in a lighter shade. Instances in which frequencies of both individuals were the same are shown in *gray* and the actual number of events is included in each cell

To provide insight into relationship dynamics within a pair, the total number of play events for each member of same-sex pairs was charted (Fig. 7). In some pairs, one partner consistently exhibited more play events, but in other pairs, there was no predictability across days in which partner played more. This was true for both female-female and male-male pairs. A measure of who was the initiator of play was gained from separate analyses of pouncing which begins with one animal. Again, in some pairs, there was a consistent initiator, and in others, there was not. Pinning is the end result of some but not all pounces. There was a tendency for one individual to pin the other more frequently. Female pair #2 exhibited an inverse relationship between pinning and pouncing, indicating an incomplete progression of the play bout. Overall, pinning was the most consistent behavior across days in both male-male and female-female dyads. Nonetheless, these data demonstrate that at the ages studied, there is a dynamic relationship in play between the partners regardless of sex.

### Sex differences in weight cannot fully account for differences in play

**Fig. 8:**
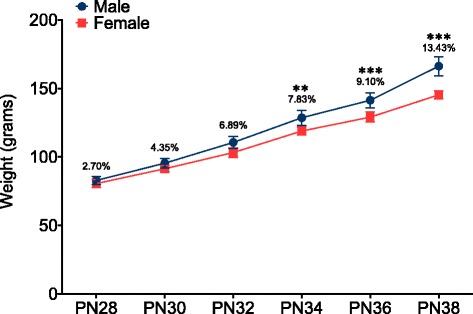
Males weigh more than females only at older ages. A repeated measures ANOVA of weight with age as a repeating factor demonstrated a significant interaction between sex and age. Bonferroni post hoc analysis revealed significant differences between males and females at PN34, PN36, and PN38 (two-way repeated measures ANOVA with Bonferroni post hoc: **p < 0.01, ***p < 0.001). The percent difference between males and females is noted above each pair of data points

Because weight differences between males and females could have contributed to sex differences observed in mixed sex pairs, weights were recorded in another group of animals every other day from PN28–PN38 and the percent difference between males and females calculated (Fig. 8). A repeated measures ANOVA, with age as the repeated factor revealed a significant interaction between age and sex (*F*(5,35) = 23.27, *p* = 0.001). Bonferroni post hoc analysis demonstrated that males weigh more than females only at older ages (PN34–PN38) (*p* < 0.01 at PN34 and *p* < 0.001 at PN36 and PN38). Because sex differences in total play frequency, frequencies of the specific parameters of play, and time spent engaged in play behavior did not show age-associated changes that correlated with weight, sex differences in play cannot be fully explained by weight differences.

## Discussion

Social play behavior is a critical experience for proper development and maturation of juveniles in many and diverse species. Uncovering and investigating variations in play behavior may aid in revealing factors that modulate social behaviors, and are therefore likely to be affected in disorders characterized by altered social interactions. Here, we have performed a detailed investigation of how sex of self and play partner can influence rough-and-tumble play, the most common form of social play behavior in rats. Our data demonstrated a higher frequency of rough-and-tumble play in males relative to females; however, there were qualitative differences in the specific parameters of play behavior that were affected by sex that were also impacted by sex of the play partner and familiarity of the play partner. Interestingly, we found that the sex and familiarity of the play partner can have different effects depending on the sex of the test subject. These data demonstrate that the sex differences in play behavior are robust but subject to change based on experimental design.

Many studies have reported a sex difference in the initiation of play behavior [20, 23, 24, 34, 40, 41]. We observed a sex difference in pouncing behavior only in mixed-sex pairs, while there were sex differences in pinning behavior in both mixed- and same-sex pairs. We scored pinning for the animal that was standing on top of the other animal, which had rotated to a supine position. Scoring in this manner is agnostic as to whether a change in pinning is due to a change in the frequency of initiation or due to a change in defensive strategy. Considering differences in pinning versus pouncing is informative. For pinning behavior, males exhibited a higher frequency when paired with other males than when paired with females. Conversely, for pouncing, males paired with females displayed higher frequencies than when paired with other males, indicating that male initiation of play was not responsible for the decreased number of pins. This suggests that the decrease in pinning observed in males paired with females was the result of the females responding differently to play solicitation from the males.

Both sexes use rotating to supine as the most common form of defense and respond with equal frequency to attacks [26, 33]. However, females tend to respond earlier to attacks, whereas males typically wait to respond until the attacker is near the nape of the neck [26]. This difference in the timing of defense is mediated by androgens [42]. A previous study demonstrated that females showed higher frequencies of complete rotations to supine than males [33]. Our results suggest that females chose a defensive strategy other than rotation to supine based on the decreased number of pins for males paired with females and for females paired with other females. Our results may differ from those previously reported [33] because our animals were not socially isolated prior to analysis or because they also experienced play with an opposite-sex partner.

For defensive strategies, the choice to use rotation over evasion is mediated by cortical regions [43, 44]. Lesions to the medial prefrontal cortex decrease initiation of attacks and increase the use of evasion over full rotation as a defensive strategy [39]. Together, the combination of reduced attack/pouncing and increased evasion, a tactic that terminates a play bout as opposed to pinning, which facilitates play, demonstrates a decreased interest in play in animals with medial prefrontal cortex lesions. Similarly, ablating the motor cortex of rats causes an increase in evasive defense tactics [44]. It is possible that sex differences in the cortical responses to steroid hormones influence sex differences in playful defense. This could also occur indirectly from the medial amygdala, which is known to modulate sex differences in social play behavior [31].

There are multiple factors that can impact the appearance of sex differences in play, including housing conditions, prior learning, body weight, and dominance hierarchies. Brief periods of social isolation are well known to increase levels of play and is a useful strategy for exploring specific parameters of play that occur at low frequency during baseline play. Many studies have not observed sex differences when employing isolation protocols, and some have speculated this may be because isolation-evoked play is at a ceiling level for both males and females [45]. Learning can reduce sex differences in play as well. For example, repeated play refusal from females can decrease male play behavior, and repeated play solicitation from males can increase female play behavior. Thor and Holloway [24] found that the sex of cagemates can affect social behavior in males, although no effect was observed on females. In our study, animals were housed in same-sex groups to minimize effects of play with the other sex outside of the testing period. However, despite the animals being housed in same-sex groups, there could be some learning or establishment of dominance hierarchies that occurred during the play sessions, particularly in the mixed-sex pairs where the weight differential would be the greatest. In our study, weight differences did not correlate with differences in play behavior and learning did not appear to be a major confound since the time spent engaged in play for the three pair types did not vary significantly over the many days of testing. However, familiarity with daily partners may have been established as evidenced by the change in female behavior with the introduction of a novel play partner. In social play behavior, the dominant individual is often determined by the individual that most frequently occupies the on-top position during pinning [46]; however, some studies report that dominance does not emerge until after sexual maturity [47]. In a study of social play with repeated pairing with the same partner, a “dominance” hierarchy was established, with one animal achieving the on-top position during pinning more often than the other; however, this was not always the heaviest individual in the pair [46]. Furthermore, experimental manipulations that increase play during the juvenile stage do not alter dominance relationships in adulthood [35]. Since “dominance” during juvenile play does not correlate with weight or adult dominance hierarchies, it is likely that what is considered “dominance” during the juvenile stage is simply elevated playfulness. Our results demonstrate that within a pair, one individual did achieve the top pinning position more frequently; however, this individual was not always the one with the highest total play frequency.

Previous studies have reported intermediate levels of play in mixed-sex pairs [26]. This may be partially due to the observation that individuals of both sexes are more likely to respond to playful initiation from a male rather than from a female, which would decrease play behavior of a male when he is paired with a female and decrease the level of play in a female paired with another female below that which is observed when that female is paired with a male [26]. In our study, intermediate levels of play in mixed-sex pairs was most noticeable in the time spent engaged in play behavior and male pinning behavior. Interestingly, there was no increase in the frequency of female play behavior when she was paired with a male versus another female, for any of the parameters. One possible explanation for the decrease in male behavior when he is paired with a female could be that males find something undesirable about the quality of play put forth by females. In other species, such as antelope calves, sheep, wallabies, vervet monkeys, and baboons, individuals display strong preferences for specific play partners [48–53]. When paired with a random partner rather than having free choice, a decrease in quality of play was observed despite the observation that there was no change in the frequency of play [52]. Time spent engaged in play is often considered a measure of the quality of play, since it does not always correlate with the frequency of play [25]. Consistent with this, an increase in time spent engaged in play behavior was the only parameter in which female play was altered when paired with a male. Alternatively, rats have been shown to decrease their play when paired with a less playful partner and to increase their play when paired with a more playful partner [26, 34–38]. This would suggest that if females are not as responsive to the males’ play initiation attempts, or if they respond in a way that is undesirable to the male, the male will eventually stop attempting to play. We, however, did not observe a decrease in the males’ attempts to initiate play when paired with females. Some possible explanations could be that our individual play sessions were relatively short, which prevented the male from reaching the point of giving up the attempt to initiate play, and because group housing and play sessions with other males in combination with the daily play sessions with females provided the males with responsive play partners to revive their interest in play.

Although female play appeared to be mostly unaffected by the sex of the play partner, there was a preference for familiar over novel play partners. For total frequency of play, we observed a decrease in play behavior on PN33 when a novel female play partner was introduced. This did not occur when the novel play partner was a male or in males when paired with a novel play partner of either sex. It is well established that rodents can discriminate between novel and familiar objects even with a delay of several days [54]; however, it is possible this is not the case for social recognition. One study demonstrated a loss of preference for a novel over familiar stimulus animal within the course of a few hours [55]. Interestingly, in this study, females retained preference for the novel stimulus longer than males, which could help to explain why we only observed a change in play behavior in female-female pairs. Ŝpinka et al. [14] suggested that animals prefer to play with a familiar over an unfamiliar partner because the unfamiliar partner could “cheat” in self-handicapping. The theory of self-handicapping suggests that during play, individuals will let the other “win” because play would not be as enjoyable for an individual that was losing constantly and would result in termination of play. Self-handicapping is observed in diverse species including domestic dogs, wallabies, and primates [17, 25, 56]. However, our observation that this only occurs in female-female pairs needs further investigation. It is also unclear why the decrease in play behavior was observed in female-female pairs on PN33 but not on the first day of play behavior at PN27. There are several possible explanations for this observation. Older animals may simply be more sensitive to a novel play partner compared to younger animals. It is also possible that the pair play recorded during our experimental sessions was more sensitive to novelty than group play, which would have been any play experienced in the home cage prior to the first day of testing. Additionally, it is unknown how much the animals engaged in play in their home cages prior to PN27, which could leave the possibility that they were not experienced enough to have established attachment to specific play partners. Further experiments would be required to determine which of these factors contributed to our results.

## Conclusions

In summary, we have observed sex differences in juvenile social play behavior indicating that there are both quantitative and qualitative differences. Interestingly, we noticed changes in male parameters of play when paired with females that were indicative of changes in female defensive strategy when approached for play by a male. We also demonstrated that males exhibit robust changes in their play behavior depending on the sex of their play partner. In contrast, females did not change their behavior in response to the sex of the play partner for most parameters. However, females did demonstrate a preference for familiar rather than novel play partners. Together, these data are useful for understanding how methodology can be critical when analyzing sex differences in social play behavior, and suggest that the most reliable sex differences can be observed in same-sex/treatment pairs so that results are not confounded by relative differences in the playfulness of the partners. Additionally, we provide a detailed analysis of which parameters of play display sex differences, providing important information to elucidate factors contributing to increased or decreased social play behaviors.

## References

[1] Arthur M, Bochner S, Butterfield N (1999). Enhancing peer interactions within the context of play. Int J Disabil Dev Educ.

[2] Graham KL, Burghardt GM (2010). Current perspectives on the biological study of play: signs of progress. Q Rev Biol.

[3] Cordoni G (2009). Social play in captive wolves (Canis lupus): not only an immature affair. Behaviour.

[4] Henry JD, Herrero SM (1974). Social play in the American black bear: its similarity to canid social play and an examination of its identifying characteristics. Integr Comp Biol.

[5] Pellis SM, Pellis VC (1997). The prejuvenile onset of play fighting in laboratory rats (Rattus norvegicus). Dev Psychobiol.

[6] Pellis SM, Pellis VC (1998). Play fighting of rats in comparative perspective: a schema for neurobehavioral analyses. Neurosci Biobehav Rev.

[7] Cordier R, Bundy A, Hocking C, Einfeld S (2010). Empathy in the play of children with attention deficit hyperactivity disorder. OTJR Occup Particip Health.

[8] Jordan R (2003). Social play and autistic spectrum disorders: a perspective on theory, implications and educational approaches. Autism Int J Res Pract.

[9] Møller P, Husby R (2000). The initial prodrome in schizophrenia: searching for naturalistic core dimensions of experience and behavior. Schizophr Bull.

[10] Strous RD, Alvir JMJ, Robinson D, Gal G, Sheitman B, Chakos M (2004). Premorbid functioning in schizophrenia: relation to baseline symptoms, treatment response, and medication side effects. Schizophr Bull.

[11] Byers JA, Walker C (1995). Refining the motor training hypothesis for the evolution of play. Am Nat.

[12] Gruendel AD, Arnold WJ (1969). Effects of early social deprivation on reproductive behavior of male rats. J Comp Physiol Psychol.

[13] Pellis SM, Pellis VC, Bell HC. The function of play in the development of the social brain. Am J Play. 2010;279–96.

[14] Spinka M, Newberry RC, Bekoff M (2001). Mammalian play: training for the unexpected. Q Rev Biol.

[15] van den Berg CL, Hol T, Van Ree JM, Spruijt BM, Everts H, Koolhaas JM (1999). Play is indispensable for an adequate development of coping with social challenges in the rat. Dev Psychobiol.

[16] Auger AP, Olesen KM (2009). Brain sex differences and the organisation of juvenile social play behaviour. J Neuroendocrinol.

[17] Leresche LA (1976). Dyadic play in hamadryas baboons. Behaviour.

[18] Olioff M, Stewart J (1978). Sex differences in the play behavior of prepubescent rats. Physiol Behav.

[19] Palagi E, Antonacci D, Cordoni G (2007). Fine-tuning of social play in juvenile lowland gorillas (gorilla gorilla gorilla). Dev Psychobiol.

[20] Parent CI, Meaney MJ (2008). The influence of natural variations in maternal care on play fighting in the rat. Dev Psychobiol.

[21] Pellis SM (2002). Sex differences in play fighting revisited: traditional and nontraditional mechanisms of sexual differentiation in rats. Arch Sex Behav.

[22] Takahashi LK, Lore RK (1983). Play fighting and the development of agonistic behavior in male and female rats. Aggress Behav.

[23] Thor DH, Holloway WR (1985). Play soliciting behavior in prepubertal and postpubertal male rats. Anim Learn Behav.

[24] Thor DH, Holloway WR (1986). Social play soliciting by male and female juvenile rats: effects of neonatal androgenization and sex of cagemates. Behav Neurosci.

[25] Watson DM, Croft DB (1993). Playfighting in captive red-necked wallabies, macropus rufogriseus banksianus. Behaviour.

[26] Pellis SM, Field EF, Smith LK, Pellis VC (1997). Multiple differences in the play fighting of male and female rats. Implications for the causes and functions of play. Neurosci. Biobehav. Rev.

[27] Daenen EWPM, Wolterink G, Gerrits MAFM, Van Ree JM (2002). The effects of neonatal lesions in the amygdala or ventral hippocampus on social behaviour later in life. Behav Brain Res.

[28] Vanderschuren LJ, Niesink RJ, Van Ree JM (1997). The neurobiology of social play behavior in rats. Neurosci Biobehav Rev.

[29] Shughrue PJ, Lane MV, Merchenthaler I (1997). Comparative distribution of estrogen receptor-alpha and -beta mRNA in the rat central nervous system. J Comp Neurol.

[30] Simerly RB, Chang C, Muramatsu M, Swanson LW (1990). Distribution of androgen and estrogen receptor mRNA-containing cells in the rat brain: an in situ hybridization study. J Comp Neurol.

[31] Meaney MJ, McEwen BS (1986). Testosterone implants into the amygdala during the neonatal period masculinize the social play of juvenile female rats. Brain Res.

[32] Olesen KM, Jessen HM, Auger CJ, Auger AP (2005). Dopaminergic activation of estrogen receptors in neonatal brain alters progestin receptor expression and juvenile social play behavior. Endocrinology.

[33] Himmler SM, Modlinska K, Stryjek R, Himmler BT, Pisula W, Pellis SM. Domestication and diversification: a comparative analysis of the play fighting of the Brown Norway, Sprague-Dawley, and Wistar laboratory strains of (Rattus norvegicus). J. Comp Psychol Wash DC 1983. 2014;128:318–27. 10.1037/a0036104 24749500

[34] Thor DH, Holloway WR (1983). Play-solicitation behavior in juvenile male and female rats. Anim Learn Behav.

[35] Pellis SM, Pellis VC, Kolb B (1992). Neonatal testosterone augmentation increases juvenile play fighting but does not influence the adult dominance relationships of male rats. Aggress Behav.

[36] Pellis SM, McKenna MM (1992). Intrinsic and extrinsic influences on play fighting in rats: effects of dominance, partner’s playfulness, temperament and neonatal exposure to testosterone propionate. Behav Brain Res.

[37] Pellis SM, McKenna M (1995). What do rats find rewarding in play fighting?--An analysis using drug-induced non-playful partners. Behav Brain Res.

[38] Reinhart CJ, McIntyre DC, Metz GA, Pellis SM (2006). Play fighting between kindling-prone (FAST) and kindling-resistant (SLOW) rats. J Comp Psychol Wash DC 1983.

[39] Bell HC, McCaffrey DR, Forgie ML, Kolb B, Pellis SM (2009). The role of the medial prefrontal cortex in the play fighting of rats. Behav Neurosci.

[40] Jessen HM, Kolodkin MH, Bychowski ME, Auger CJ, Auger AP (2010). The nuclear receptor corepressor has organizational effects within the developing amygdala on juvenile social play and anxiety-like behavior. Endocrinology.

[41] Kurian JR, Bychowski ME, Forbes-Lorman RM, Auger CJ, Auger AP (2008). Mecp2 organizes juvenile social behavior in a sex-specific manner. J Neurosci Off J Soc Neurosci.

[42] Pellis SM, Pellis VC, McKenna MM. Feminine dimension in the play fighting of rats (Rattus norvegicus) and its defeminization neonatally by androgens. J Comp Psychol Wash DC 1983. 1994;108:68–73. 10.1037/0735-7036.108.1.68 8174346

[43] Bell HC, Pellis SM, Kolb B (2010). Juvenile peer play experience and the development of the orbitofrontal and medial prefrontal cortices. Behav Brain Res.

[44] Kamitakahara H, Monfils M-H, Forgie ML, Kolb B, Pellis SM (2007). The modulation of play fighting in rats: role of the motor cortex. Behav Neurosci.

[45] Thor DH, Holloway WR (1984). Sex and social play in juvenile rats (Rattus norvegicus). J Comp Psychol.

[46] Panksepp J (1981). The ontogeny of play in rats. Dev Psychobiol.

[47] Adams N, Boice R (1989). Development of dominance in domestic rats in laboratory and seminatural environments. Behav Processes.

[48] Berger J (2009). The ecology, structure and functions of social play in Bighorn sheep (Ovis canadensis). J Zool.

[49] Cheney DL (1978). The play partners of immature baboons. Anim Behav.

[50] Gomendio M (1988). The development of different types of play in gazelles: implications for the nature and functions of play. Anim Behav.

[51] Govindarajulu P, Hunte W, Vermeer LA, Horrocks JA (1993). The ontogeny of social play in a feral troop of vervet monkeys (Cercopithecus aethiops sabaeus): the function of early play. Int J Primatol.

[52] Thompson KV (1996). Play-partner preferences and the function of social play in infant sable antelope, Hippotragus niger. Anim Behav.

[53] Watson DM (2010). The play associations of red-necked wallabies (Macropus rufogriseus banksianus) and relation to other social contexts. Ethology.

[54] Antunes M, Biala G (2012). The novel object recognition memory: neurobiology, test procedure, and its modifications. Cogn Process.

[55] Markham JA, Juraska JM (2007). Social recognition memory: influence of age, sex, and ovarian hormonal status. Physiol Behav.

[56] Bauer EB, Smuts BB (2007). Cooperation and competition during dyadic play in domestic dogs, Canis familiaris. Anim Behav.

